# Determinants of utilisation rates of preventive health services: evidence from Chile

**DOI:** 10.1186/s12889-018-5763-4

**Published:** 2018-07-06

**Authors:** Elena S. Rotarou, Dikaios Sakellariou

**Affiliations:** 1 Department of Economics, University of Chile, Diagonal Paraguay 257, Office 1506, 8330015 Santiago, Chile; 2 Cardiff University, School of Healthcare Sciences, Eastgate House, Newport Road 35-43, Cardiff, CF24 0AB UK

**Keywords:** Preventive health services, Healthcare, Public health provider, Private health provider, Chile, Health inequality

## Abstract

**Background:**

Preventive health services play a vital role in population health. However, access to such services is not always equitably distributed. In this article, we examine the barriers affecting utilisation rates of preventive health services, using Chile as a case study.

**Methods:**

We conducted a cross-sectional study analysing secondary data from 206,132 Chilean adults, taken from the 2015 National Socioeconomic Characterisation Survey of the Government of Chile. We carried out logistic regressions to explore the relationship between the dependent variable *use of preventive services* and various demographic and socioeconomic variables.

**Results:**

Categories more likely to use preventive services were women (OR=1.16; 95%CI: 1.11–1.21) and inactive people (OR=1.41; 95%CI: 1.33–1.48). By contrast, single individuals (OR= 0.85 ; 95%CI: 0.80–0.91) and those affiliated with the private healthcare provider (OR= 0.89; 95%CI: 0.81–0.96) had fewer odds of undertaking preventive exams.

**Conclusions:**

The findings underline the necessity of better information campaigns on the availability and necessity of preventive health services, addressing health inequality in accessing health services, and tackling lifestyle-related health risks. This is particularly important in countries – such as Chile – characterised by high income inequality and low utilisation rates of preventive health services.

## Background

Global demographic, epidemiological, and socioeconomic changes – such as ageing, urbanisation, globalisation, and reductions in morbidity and mortality rates – have resulted in the increase in the prevalence of chronic diseases, and therefore, have underlined the need for effective strategies that will improve global health [1]. As a result, preventive health services have been introduced in many countries, since studies have shown that provision of such services can lead to health management in the early stage of diseases, and that prevention can contribute to reducing the subsequent total demand for medical care [2].

In this article, we investigate the utilisation rates of preventive health services for Chilean adults (people of 15 years of age and over) and the various demographic, socioeconomic, and health-related factors influencing such rates. We focus particularly on differences in utilisation rates on the basis of affiliation with the public or private health provider. The importance of this study lies on the fact that Chile, as many other developed countries, has been experiencing an ageing of its population: the over-60 group – currently 11.1% of the population – is expected to reach 13% by 2020, underlining the increasing need for preventive services, as population becomes more likely to suffer from chronic and age-related diseases [3]. Despite the growing importance of preventive services, there are currently very few studies that have investigated preventive health services in Chile (for instance, Baechler, Barra and Soto, on preventive medicine index for the region of Maule; Sakellariou and Rotarou, on utilisation of cancer screening services by disabled women) [4, 5].

Unlike many international studies that have used specific subpopulations and health surveys to investigate utilisation of preventive health services and the factors affecting such rates (for example, Schülein et al., on participation in preventive health check-ups of German women; Yen et al., on use of preventive services among adults with intellectual disabilities in Taiwan) [6, 7], in this study we use cross-sectional data from over 200,000 Chilean adults from the general population, available from the National Socioeconomic Characterisation Survey of the Government of Chile.

This article contributes to existing literature by investigating the influence of health care provider on the utilisation rates of preventive services. In the context of highly unequal access to health care services, and neoliberal practices in the Chilean health system [8–10], such an investigation is very important, since it can guide policy makers in the design, implementation, and monitoring of strategies, which will address not only the availability of preventive health services but also possible obstacles in the effective access and utilisation of such services. Overall, this study addresses a gap in existing literature, both in terms of contribution to general knowledge on utilisation rates and possible barriers affecting preventive health services, but also in terms of knowledge about access and utilisation of preventive exams in Chile, a country characterised by high income inequality and stratification of its health care services.

We first give a brief presentation of the health system and preventive health services in Chile, before moving on to the methodology and study results, which we critically discuss in tandem with relevant literature.

## Health system and preventive health services in Chile

The health coverage in Chile is universal, that is, all citizens are entitled to access and utilisation of health care services. The public health care provider (Fondo Nacional de Salud, FONASA) is divided into four segments (A, B, C, and D), according to individual or family income; very poor people or people without any income belong to segment A and receive treatment free of charge. In 2016, most Chileans (74.4%) were affiliated with FONASA, while 18.7% of the population were affiliated with one of the private health care providers (Instituciones de Salud Previsional, ISAPREs); the remaining population either paid out-of-pocket or was affiliated with the health provider of the Armed Forces [11].

The separation of the health system in Chile into public and private providers – which took place during the late 1970s and early 1980s – led to the creation of health inequalities through the stratification of access and utilisation of health care services [8, 10, 12]. Thus, people from higher socioeconomic classes are usually affiliated with an ISAPRE, and enjoy better-quality health care services and timely attention, while people from lower socioeconomic classes access FONASA, which is usually characterised by much longer waiting times and poorer infrastructure. The contract premium for the ISAPREs is determined by age, sex, and risk factor, a fact that often excludes the elderly and women of reproductive age [12]. For example, in 2015 only 11.7% of total ISAPRE affiliates were people over 60 years of age, and only about 35% were women [13]. A recent study by the Superintendence of Health revealed that a 35-year-old woman can pay 66% more on average for her ISAPRE plan than a 35-year-old man [14].

Concerning preventive health services, the Preventive Medical Examination (PME) was established in Chile as a public health policy, and was defined by the Superintendence of Health as a periodic, voluntary, and free health evaluation [15]. All individuals affiliated with either the public health care provider (FONASA) or the private providers (ISAPREs) are entitled to use preventive exams free of charge themselves and their family, depending on their perceived health risk and age [16]. This PME is a periodic and voluntary health monitoring and evaluation plan that forms part of the Explicit Health Guarantees for Chileans benefits package (Acceso Universal a Garantías Explícitas en Salud- Garantías Explícitas en Salud, AUGE-GES). The AUGE-GES plan establishes explicit guarantees for people in a group of eighty prioritised pathologies (as of July 2017), independent of their ability to pay for health services and treatment.

The available preventive health services for adults (people aged 15 and older) in Chile include the following: screening and early detection of alcohol misuse (identification of risk behaviours and prevention options), identification of smoking problem (through medical history and/or test of Fagerström to check level of smoking addiction), identification and treatment of overweight and obesity, as well as various tests to identify, measure and/or assess arterial hypertension, diabetes mellitus, syphilis, and tuberculosis. Women aged 25–64 are entitled to Papanicolaou cervical smear test to screen for cervical cancer, and women aged 50–69 to a mammography for breast cancer. Adults of 40 years of age and older can undertake dyslipidaemia controls, and people of 65 years of age and older can undergo various screening tests to assess their functional independence [17]. These preventive health checks are available free of charge only under the following circumstances: a) if individuals are affiliated with FONASA, they can ask for the corresponding health services at the health facility where they are registered; and b) if individuals are affiliated with an ISAPRE, they can have preventive services only at the health facilities and with the doctors with which their ISAPRE has a plan [16].

In 2015, there were 1,605,219 Chilean adults (about 11.2% of the total population of 15 years of age and older) that used preventive services, out of which 43.4% were male and 56.6% female [18]. This number reflects only preventive health services performed in the public health system. Regarding preventive health checks in the private system, the information is quite scarce. Preventive checks in private hospitals or clinics in the capital, Santiago, have increased in the last five years by 30%; between 1500 and 6500 people undergo such exams every year at each private hospital, with the majority being men with an average age of 50 [19]. Taking into account this information, a rough estimate of the percentage of Chileans adults that use preventive services every year in the country would put it at about 13–14% of the total adult population (about 14 million in 2015).

## Methods

### Study aims

The main aim of the study was to examine the utilisation rates of preventive health services for Chilean adults in 2015, and the demographic, socioeconomic, and health-related factors influencing such utilisation. We also explored whether affiliation with the public or private health care provider influenced the utilisation rates of preventive services.

### Survey and methods

The study was based on a secondary analysis of cross-sectional data available from the 2015 National Socioeconomic Characterisation Survey (Encuesta Nacional de Caracterización Socioeconómica – CASEN). CASEN is a survey conducted by the Ministry of Social Development of the Government of Chile every two to three years, since 1990. It is the main socioeconomic measurement instrument for the design and the evaluation of existing social policies in the country [20]. Its aim is the estimation of the magnitude of poverty and income distribution, the identification of the needs of the population, and the evaluation of the gaps that separate the different social segments and geographical areas. In order to achieve these goals, the survey is comprised of seven modules: Residents Registry, Education, Employment, Income, Health, Residents, and Housing [21].

The CASEN survey allows for up-to-date diagnoses on the situation of disadvantaged groups that are social policy priorities, such as children and adolescents, old people, indigenous people, and people with disabilities. It also offers information on a variety of other relevant social issues, such as access to information and communications technologies, and social participation; since 2015, it provides information on sexual diversity, the environment, and networks available to households [20].

The units of analysis (households and people) are selected in a probabilistic, stratified, and multistage manner, with the sample being representative at country level, geographical area (urban and rural), regional level (fifteen country regions), and municipal level (324 municipalities). The CASEN survey is a valid and reliable measure of reference, since the information provided there is consistent with that provided by other data sources, such as population censuses [22].

The 2015 CASEN survey covered 83,887 households, and a total of 266,968 people. Personal interviews were employed – lasting, on average, 47 min for a household of four people – from November 2nd 2015 until January 31st 2016. An interviewer with a questionnaire on paper interviewed the head of the household or a member of the household older than 18 years of age. No personal information, such as ID number or names, was requested during the interview. The microdata were fully anonymised and in the public domain (accessible from http://observatorio.ministeriodesarrollosocial.gob.cl/casen/casen_2015.php), and therefore no ethical approval was required by the Universidad de Chile.

Logistic regressions were used to investigate the demographic, socioeconomic, and health-related factors that affected the utilisation rates of preventive health services of Chilean adults. Estimated probabilities of using preventive services, depending on health provider affiliation, were also calculated. We used STATA/MP version 14.2 for all calculations.

### Data and variables

The sample size of the study included 206,132 observations (people younger than 15 years of age were excluded). People were asked whether they had undergone any health controls (with a subsequent list of various health checks). When people answered ‘yes’ to having used preventive health services, this answer was left as ‘yes’; when people answered that they had not done any health exams or they had done other type of exams (for example, dental exams or controls of chronic illnesses), this answer was left as ‘no’. As a result, the dependent variable ‘use of preventive services’ is a binary variable with answers ‘no’ and ‘yes’.

The demographic, socioeconomic, and health-related variables that were used as controls in the study included the following: a) *sex*: man / woman; b) *geographical location*: urban / rural; c) *age*: years (people 15 years of age and older); d) *civil status*: married / living with someone or in a relationship / separated, divorced or annulled / widowed / single; e) *indigeneity*: not indigenous / indigenous (includes people from nine state-recognised indigenous groups); f) *nationality*: Chilean (Chilean or with double nationality, one of them being Chilean) / foreigner; g) *health self-assessment*: scores 1–2 = ‘bad’ / scores 3–5= ‘neither good nor bad’ / scores 6–7 = ‘good’; h) *health provider*: FONASA (public) / Armed forces / ISAPRE (private) / out-of-pocket; i) *disability*: no disability / with disability; j) *equalised income* (log): household income divided by square root of household size (square root equivalence scale); k) *education*: years of schooling; and l) *employment*: employed / unemployed / inactive.

## Results

### Descriptive statistics

Table 1 summarises the characteristics of the study sample, revealing that more women than men use preventive health services (61% vs. 39%), in agreement with relevant literature [23, 24]. The average age of people using preventive services in our sample is 67, with most of them (47%) being married. More people that use preventive services are affiliated with FONASA (89%) and fewer are affiliated with an ISAPRE (7.4%), compared to people not using such services (82 and 12.4%, correspondingly). As expected, a higher percentage (59%) of people not using preventive health services assess their health as ‘good’, while only 37% of people using such services do so; this may be related to the higher percentage of people using preventive services that have some kind of disability. Concerning socioeconomic status, more people not using preventive services are employed, earning a slightly higher income, and having more years of education, compared to people who do use such services.

**Table 1:** Demographic, socioeconomic, and health-related characteristics of the sample

Parameter		2015 (*n* = 206,132)	
		No preventive services (n, %)	Preventive services (n,%)
Sex	Male	91,771 (47.1%)	4415 (38.9%)
	Female	103,021 (52.9%)	6925 (61.1%)
	Chi-square test *p*-value	*p* < 0.0001	
	Cramer’s V	0.037	
Zone	Urban	151,961 (78.0%)	8746 (77.1%)
	Rural	42,831 (22.0%)	2594 (22.9%)
	Chi-square test *p*-value	*p* = 0.027	
	Cramer’s V	0.005	
Age (mean, std. dev.)		42.9 (18.5)	67.0 (15.9)
	t-test *p*-value	*p* < 0.0001	
	Point biserial correlation	−.286	
	Married	67,871 (34.8)	5319 (46.9%)
	Living with or in a relationship	31,351 (16.1%)	921 (8.1%)
Civil status	Separated, divorced, annulled	13,043 (6.7%)	905 (8.0%)
	Widowed	9958 (5.1%)	2648 (23.4%)
	Single	72,569 (37.3%)	1547 (13.6%)
	Chi-square test *p*-value	*p* < 0.0001	
	Cramer’s V	0.202	
Nationality	Chilean	191,779 (98.5%)	11,263 (99.3%)
	Foreigner	3013 (1.6%)	77 (0.7%)
	Chi-square test *p*-value	*p* < 0.0001	
	Cramer’s V	−0.016	
Indigeneity	Not indigenous	173,670 (89.2%)	10,356 (91.3%)
	Indigenous	21,122 (10.8%)	984 (8.7%)
	Chi-square test *p*-value	*p* < 0.0001	
	Cramer’s V	−0.016	
Health system	FONASA	159,586 (81.9%)	10,092 (89.0%)
	Armed forces	4190 (2.2%)	325 (2.9%)
	ISAPRE	24,210 (12.4%)	833 (7.4%)
	Out-of-pocket	6806 (3.5%)	90 (0.8%)
	Chi-square test *p*-value	*p* < 0.0001	
	Cramer’s V	0.052	
Health score	Bad	13,036 (6.7%)	1487 (13.1%)
	Average	67,685 (34.8%)	5632 (50.0%)
	Good	114,071 (58.6%)	4221 (37.2%)
	Chi-square test *p*-value	*p* < 0.0001	
	Cramer’s V	0.102	
Disability	No disability	175,756 (90.2%)	8434 (74.4%)
	With disability	19,036 (9.8%)	2906 (25.6%)
	Chi-square test *p*-value	*p* < 0.0001	
	Cramer’s V	0.117	
Employment	Employed	103,565 (53.2%)	3176 (28.0%)
	Unemployed	8423 (4.3%)	156 (1.4%)
	Inactive	82,804 (42.5%)	8008 (70.6%)
	t-test *p*-value	*p* < 0.0001	
	Cramer’s V	0.130	
Equalised income^a^ (mean, std. dev.)		528,348 (671,874)	509,400 (549,153)
	t-test *p*-value	*p* = 0.003	
	Point biserial correlation	.006	
Education, (mean, std. dev.)		10.6 (4.2)	7.8 (4.9)
	t-test *p*-value	*p* < 0.0001	
	Point biserial correlation	.151	

^a^ Chilean pesos (1 USD = 659 Chilean pesos, average July 2017)

**Fig. 1:**
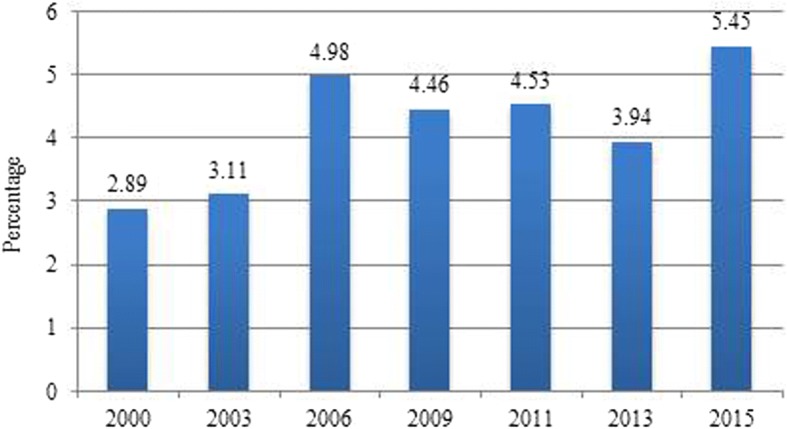
Chilean adults (%) using preventive health services, 2000–2015

Figure 1 shows the percentage of Chilean adults that underwent preventive services during the period 2000–2015. It should be noted that in the pre-2011 CASEN surveys, the general question on health controls and on preventive health services was slightly different: this can be reflected especially in the low percentage of people answering that they have used preventive services for the years 2000 and 2003.

According to the 2015 data, 5.5% of Chilean adults used preventive health services, the highest percentage since the year 2000. The differences between years are statistically significant with *p* < 0.0001, with the exception of 2009–2011 (*p* = 0.274). Concerning the vast majority of preventive health services for 2015, these were performed in public hospitals or public health centres; less than 9% of preventive services were performed in private medical centres. Regarding payment, the vast majority of people who used preventive services did not pay for them, mostly because they were affiliated with the public health provider.

### Logistic regressions

Logistic regressions were employed in order to investigate the impact of various demographic, socioeconomic, and health-related factors on utilisation of preventive health services of Chilean adults. The mean variance inflation factor for all variables was 1.63, indicating that there were no collinearity issues. The results of the logistic regressions are presented in Table 2. Model (1) shows odds ratios adjusted for demographic variables, Model (2) introduces socioeconomic variables, while Model (3) adds health-related variables (fully-adjusted odds ratios, all the variables of Table 1 included). A higher Mac Fadden *R*^2^, and lower deviance, and AIC and BIC values, show that Model (3) provides a better fit than Models (1) and (2).

**Table 2:** Results of logistic regressions using *preventive services* as dependent variable

Variables	(1)	(2)	(3)
	OR (95% CI)	OR (95% CI)	OR (95% CI)
Sex (male as reference)			
Female	1.28^***^ (1.23–1.33)	1.17^***^ (1.12–1.23)	1.16^***^ (1.11–1.21)
Geographical location (urban as reference)			
Rural	.92^***^ (.87–.96)	.90^***^ (.86–.95)	.90^***^ (.86–.95)
Age (years)	1.07^***^ (1.07–1.08)	1.07^***^ (1.06–1.07)	1.07^***^ (1.06–1.07)
Civil status (married as reference)			
Living with or in a relationship	.87^***^ (.81–.93)	.89^**^ (.82–.95)	.88^**^ (.82–.95)
Separated, divorced, annulled	.96 (.89–1.04)	1.02 (.95–1.10)	1.03 (.95–1.11)
Widowed	.94^*^ (.89–.99)	.94^*^ (.88–.99)	.94^*^ (.89–.99)
Single	.85^***^ (.80–.90)	.84^***^ (.79–.89)	.85^***^ (.80–.91)
Indigeneity (not indigenous as reference)			
Indigenous	1.03 (.96–1.10)	1.03 (.96–1.10)	1.02 (.95–1.10)
Nationality (Chilean as reference)			
Foreigner	.90 (.71–1.13)	.91 (.72–1.15)	.97 (.76–1.23)
Equalised income (log)		1.11^***^ (1.08–1.15)	1.13^***^ (1.09–1.17)
Education (years)		.99^***^ (.98–.99)	.99^**^ (.98–.99)
Employment (employed as reference)			
Unemployed		1.04 (.88–1.22)	1.06 (.90–1.26)
Inactive		1.40^***^ (1.33–1.48)	1.41^***^ (1.33–1.48)
Health self-assessment (bad as reference)			
Neither good nor bad			1.18^***^ (1.11–1.26)
Good			1.14^**^ (1.06–1.22)
Health provider (FONASA as reference)			
Armed forces			.92 (.81–1.05)
ISAPRE			.89^**^ (.81–.96)
Out-of-pocket			.42^***^ (.33–.51)
Disability (no as reference)			
Yes			1.04 .99–1.10)
LR chi2	17,770.10	17,923.71	17,576.60
Prob > chi2	0.0000	0.0000	0.0000
McFadden R2	0.197	0.200	0.200
Deviance	72,250.482	71,762.361	70,243.872
AIC	72,270.482	71,790.361	70,283.872
BIC	72,373.157	71,934.053	70,488.597

^*^ *p* < 0.05, ^**^ *p* < 0.01, ^***^ *p* < 0.001

The results show that there is a gender difference, with women having 1.2 times (CI 95%: 1.11–1.21) higher odds of using preventive services than men. People living in rural areas had 1.1 times (CI 95%: .86–.95) fewer odds of undertaking preventive exams than people living in urban areas. Older people had higher odds of undergoing preventive services: one extra year increased the odds by 1.07 (CI 95%: 1.06–1.07). Concerning civil status, being married was the status associated with a higher probability of using preventive services, while single people had the lowest probability of doing so (OR: .85, CI 95%: .80–.91).

Furthermore, people with higher incomes had slightly higher odds of undertaking preventive exams than poorer people (OR: 1.13, CI 95%: 1.09–1.17). Regarding employment, people that were inactive had 1.4 times (CI 95%: 1.33–1.48) higher odds of undergoing preventive services. People with a good or an average self-assessed health score had 1.1 times (CI 95%: 1.06–1.22) to 1.2 times (CI 95%: 1.11–1.26) higher odds of undertaking preventive health checks than people who graded their health as ‘bad’. People affiliated with an ISAPRE had 1.1 times (CI 95%: .81–.96) fewer odds of undergoing preventive checks; people paying out-of-pocket had 2.4 times (CI 95%: .33–.51) fewer odds of doing so, compared with people affiliated with FONASA.

More years of education did not have an impact on utilisation of preventive services; while statistically significant, the ratio approached 1 (OR: .99, CI 95%: .98–.99), indicating no relationship. There was no statistically significant relationship between the remaining variables (that is, ‘indigeneity’, ‘nationality’, and ‘disability’) and utilisation rates of preventive health services.

**Fig. 2:**
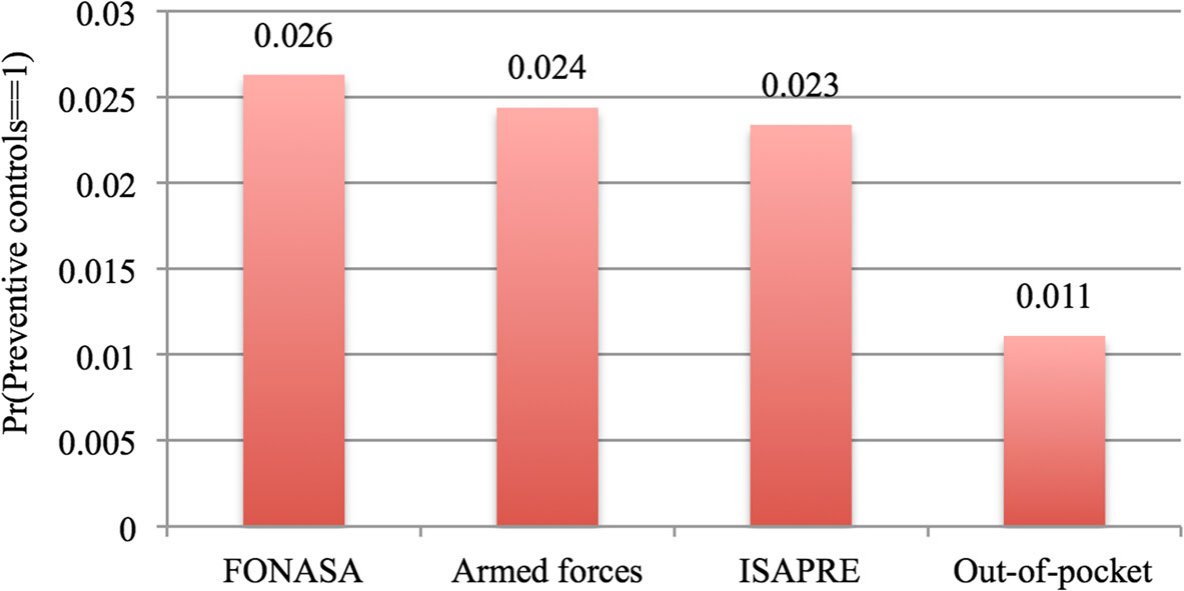
Estimated probabilities for utilisation of preventive health services with *health provider* as predictor variable

Figure 2 presents the estimated probabilities for utilisation of preventive health services when the predictor variable is *health provider*, while holding the other predictor variables at their mean.

As it can be observed in Fig. 2, Chilean adults affiliated with FONASA had a higher probability of using preventive health services, followed by people affiliated with the Armed Forces’ health care provider, and people affiliated with an ISAPRE. Chileans paying out-of-pocket had the lowest probability of using preventive health services. All predicted probabilities were significantly different from zero (*p* < 0.0001).

## Discussion

This study investigated the utilisation rates of preventive health services for Chilean adults for 2015. It also looked at differences in utilisation rates due to affiliation with the public or private health care provider. Confirming the study’s hypothesis, women were more likely to undergo preventive checks than men. This result agrees with previous studies [23, 25–28]. There are also a few studies that have shown no sex-specific difference in relation to preventive health services [24].

The findings also showed that older people had slightly higher odds of using preventive services than younger people. Research on age and use of preventive health services is quite diverse: some have shown an inverted U-turn relationship between age and preventive services [24], others a linear relationship with regards certain preventive checks, such as blood pressure checks [25], other studies have shown country differences [29, 30], while others have shown no significant age differences in utilisation rates of preventive services [31].

Concerning civil status, being married was associated with a higher probability of using preventive services than all other statuses. This result agrees with previous research indicating that generally marriage acts positively for the promotion of good health-related behaviours [32], and the increase in utilisation rates of preventive health services [33], particularly for men [34].

Furthermore, people with higher incomes were slightly more likely to use preventive health services than people with lower incomes. Literature has either indicated a positive relationship between higher income / social class and utilisation rates of preventive services [24, 35], or no relationship at all [27]. More research is necessary into the role of income and socioeconomic status in the uptake of preventive health checks.

People with an average or good self-assessed health score were more likely to undertake preventive health checks than people who graded their health as ‘bad’. Previous studies on the relationship between self-evaluated health and preventive services have not provided definite results. For example, in a study by Labeit, Peinemann, and Baker, while poor self-rated health status increased the utilisation of blood pressure checks by about 12.6%, there was no significant influence of poor health status on utilisation rates with regards to breast and cervical cancer screening [27]. Another study showed that individuals that were in poorer health were more likely to get flu shots and cholesterol checks, but less likely to have mammograms, pap smears, breast examinations, and prostate checks [36].

Being unemployed (i.e. people who are not employed but are in search of a job) did not show a statistically significant relationship with preventive services. Being inactive (i.e. people who are neither employed, nor are looking for a job, either due to age or serious disability), however, did: inactive people were 1.4 times more likely to use preventive services than employed people. This might be on account of more available free time to perform such checks. Being employed has been found to be associated with lower uptake of breast screening in the UK [27].

Finally, education did not have an impact on the utilisation rates of preventive services; while statistically significant, the coefficient was very close to 1. Taking into account that higher education is generally linked to better health outcomes and better health literacy [37], it would be reasonable to assume that people with higher education are more likely to use preventive health services. However, research on this relationship has not provided a definite answer: some studies have found a positive relationship between higher education and utilisation of preventive services [38, 39], ,others have not found a statistically significant relationship [6], while others have found a statistically significant relationship only for certain types of preventive services, such as dental screening [27].

Our findings also show that individuals affiliated with FONASA are more likely to use preventive health services than individuals affiliated with an ISAPRE. As seen previously, in order to access these services for free, FONASA affiliates have to go to the health facility where they are registered, while affiliates with an ISAPRE have to go to the providers defined by their ISAPRE; otherwise they have to pay [16]. However, ISAPRE affiliates, who usually enjoy a better socioeconomic status, often prefer going to their own doctors and health facilities, and therefore, take on an additional cost for their decision, thus paying for convenience. For example, in 2015 only 27% of the preventive services undertaken by ISAPRE affiliates were performed completely free of charge; the vast majority paid a co-payment of a total of 3.8 billion Chilean pesos (about US$ 5.8 million, current prices). The high amount of co-payments can be also due to the lack of information from the ISAPREs regarding the free preventive services to which their affiliates are entitled [40]. Employment status might be another possible reason why people affiliated with an ISAPRE do not use preventive services, since this may mean losing a day of work.

The general low percentage of Chilean adults undertaking preventive health services – estimated at around 13–14% of the total population – is a matter of concern. In the US, for example, about 20% of people use preventive health services every year, with utilisation rates depending on region and insurance type [41]. Another study showed that 50% of Austrian adults have at least one comprehensive preventive health check-up a year [24].

In the particular case of Chile, it is important that people affiliated with an ISAPRE are able to use preventive services at a clinic or hospital of their choice when they sign up with a particular ISAPRE. At the moment, only about 7% of people using preventive health services are affiliated with an ISAPRE; one of the reasons might be that they can go only to the providers defined by their ISAPRE in order to access these services free of charge.

### Strengths and limitations

Due to the cross-sectional nature of the data, we cannot make any causal inferences as to the reasons for the observed differences in the utilisation rates of preventive health services. Another limitation is that it was not possible to investigate barriers to utilisation rates of preventive services going further back in time, due to the fact that the wording of the particular question on preventive services was different pre-2011. Also, it would have been interesting if other variables could be added – such as weight or lifestyle habits – that could have had an impact on utilisation rates of preventive health services; such information, however, is not available in the CASEN survey.

This study has, nevertheless, offered interesting insights on preventive health services for adults. The literature on this topic is quite limited, with most studies investigating relatively small population samples, and focusing on particular subpopulations [7], or on specific preventive services [5]. The current study used data from over 200,000 Chilean adults – a sample that was representative of the population – taken from a valid and reliable socioeconomic survey, in an effort to investigate demographic, socioeconomic, and health-related factors influencing the utilisation rates of preventive health services.

Another significant aspect of the study is that it looked into the difference in utilisation rates, depending on the affiliation with a public or private health provider; this is particular important, especially in countries – such as Chile – that are characterised by high income inequality (Gini index of 0.485 in 2015, according to the Ministry of Social Development) [42], as well as high inequality in access and utilisation of health care services [8, 9].

## Implications and recommendations

It is necessary to address the barriers that prevent a high percentage of the population from using preventive health services. It is paramount to promote a more effective information campaign on the availability and necessity of undertaking preventive health services, especially at parts of the population who report low use. Also, the availability of primary care physicians is important, since studies have shown that this can increase people’s probability of having a physician, which in turn increases preventive health care service utilisation [43]. This should be coupled with a more active physician promotion of preventive health services [44], and a better health care provider – patient communication [45], factors that have been shown to lead to an increase in the uptake of preventive health exams. It is also important to address the wider, socio-political issues related to the uptake of preventive health services, such as affordability, accessibility, and availability of services.

## Conclusions

This study investigated the factors associated with the utilisation rates of preventive health care services for Chilean adults. The results showed that women, older people, married people, those with higher incomes, inactive people, and people with an average or good self-assessed health had higher odds of undergoing preventive checks. On the other hand, people living in rural areas, single people, and people that were affiliated with the private health care provider or paid out-of-pocket had fewer odds of doing so.

Not using preventive services can have detrimental effects on individuals and population health – particularly in countries such as Chile that are characterised by high income inequality and stratification of health care services – since it can a) lead to premature death, disability, and ill health; b) result in more days of work lost and subsequent productivity losses, as well as lost taxes and increased welfare payments; and c) lead to increased direct costs to health care systems [46]. With population ageing and an increase in age-related and lifestyle-related diseases, it is important that a larger percentage of the population has access to preventive health services.

## Abbreviations

AUGE-GES: Acceso Universal a Garantías Explícitas en Salud- Garantías Explícitas en Salud
CASEN: Encuesta Nacional de Caracterización Socioeconómica
FONASA: Fondo Nacional de Salud
ISAPRE: Instituciones de Salud Previsional
PME: Preventive Medical Examination
